# Case Report: A case of Cronkhite-Canada syndrome associated with sigmoid colon cancer and early esophageal cancer

**DOI:** 10.3389/fmed.2026.1778538

**Published:** 2026-04-29

**Authors:** Yumeng Wang, Wenyan Sun, Dingxin Wang, Conghui Cai, Qicheng Fan, Zhihang Huang, Yun Bai

**Affiliations:** 1 Graduate School, Hebei North University, Zhangjiakou, China; 2 Department of Geriatric Gastroenterology, Hebei General Hospital, Shijiazhuang, China; 3 Graduate School, North China University of Science and Technology, Tangshan, China

**Keywords:** Cronkhite-Canada syndrome, early esophageal cancer, esophageal cancer (EC), sigmoid colon cancer, synchronous neoplasms

## Abstract

This article reports a case of a patient with Cronkhite-Canada syndrome combined with sigmoid colon cancer and early esophageal cancer. In this case, CCS was diagnosed simultaneously with the discovery of sigmoid colon cancer and early esophageal cancer. After radical resection of sigmoid colon cancer, the patient received hormone therapy for 12 months. The symptoms such as diarrhea, hair loss, and malnutrition were relieved, and the gastric-duodenal and colonic polyp-like lesions were reduced. The early esophageal cancer was followed up for 3 years, but no active treatment was given for the early esophageal cancer. It is rare for both colon cancer and early esophageal cancer to occur simultaneously on the basis of CCS. To improve the understanding of the diagnosis and treatment of this disease and postoperative management, the case is reported as follows.

## Introduction

Cronkhite-Canada syndrome (CCS) is a rare non-genetic disorder characterized by multiple polyps in the digestive tract and ectodermal changes (hair loss, nail and finger/foot nail malnutrition, skin pigmentation). It is often accompanied by diarrhea, loss of appetite, and malnutrition. The incidence of this disease is approximately one in a million. The cause is still unclear and may be related to immune abnormalities, chronic inflammation, and gene mutations. Diagnosis mainly relies on typical clinical manifestations, diffuse polypoid lesions under endoscopy, and pathological examination. Currently, glucocorticoids are the main treatment method. Some patients may need to combine immunosuppressants or endoscopic and surgical treatments. CCS is closely related to colorectal cancer. A survey in Japan indicated that the prevalence of colorectal cancer in this population was as high as 10–20%, significantly higher than that of the general population. However, CCS combined with esophageal cancer is extremely rare, and only a few reports have been published domestically and internationally. This article reports a case of a 76-year-old male with CCS, in which both sigmoid colon cancer and early esophageal cancer were discovered at the time of diagnosis. After surgery and hormone therapy, the symptoms of colon cancer and CCS were relieved, but the early esophageal cancer progressed during follow-up due to lack of active treatment. This case provides an important reference for clinical understanding of the diagnosis, treatment, and follow-up management of CCS combined with multiple sites of digestive tract tumors.

## Case presentation and management

**FIGURE 1:**
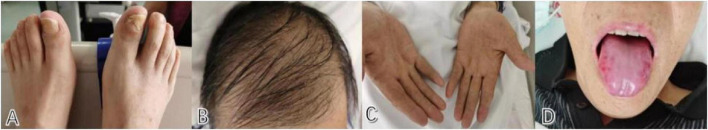
The patents body changes after onset of the disease. **(A)** The fingernails of the both toes became thin and exfoliated. **(B)** Hai loss. **(C)** The palms were hyperpigmented. **(D)** The tongue was hyperpigmented.

The patient is a 76-year-old male. He was admitted to the hospital on January 5, 2021 due to “loss of appetite and diarrhea for over a month.” His appetite has decreased by approximately 3/4 compared to before. He has 3–4 bowel movements per day, consisting of loose yellow stools with mucus and pus. Since the onset of the disease, his weight has decreased by 5 kg. The nails on both feet toes have become thinner and fallen off. In the past month, he has experienced significant hair loss, abnormal taste, and pigmentation on both palms and tongue (Figure 1). A local gastroscopy showed: “chronic gastritis, multiple polyps in the gastric and duodenal bulb, and duodenal inflammation.” The patient has a history of coronary heart disease, old myocardial infarction, paroxysmal atrial fibrillation, and type 2 diabetes. He has been regularly treated with “simvastatin 20 mg, once a day; metoprolol 25 mg, twice a day; metformin 0.25 g, three times a day.” Deny a history of alcohol consumption and smoking, and deny a family history of digestive tract tumors and genetic diseases. Physical examination upon admission: body temperature 36.5°C, heart rate 88 beats per minute, respiratory rate 18 breaths per minute, blood pressure 159/85 mmHg. No abnormalities were found in the physical examination of the heart, lungs, and abdomen. Laboratory tests: albumin 33.6 g/L.

**FIGURE 2:**
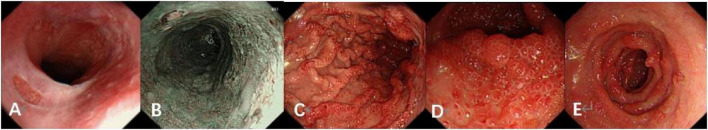
Gastroscopy after admission (2021-01-07). **(A,B)** Esophageal mucosal lesions. **(C,D)**. The mucosa of the gastric body and cardia was diffusely congested and edematous, accompanied by multiple polypaid lesions. The polyps varied in size and were distributed in clusters. **(E)** Gastric and duodenal mucosal polypaid changes.

**FIGURE 3:**
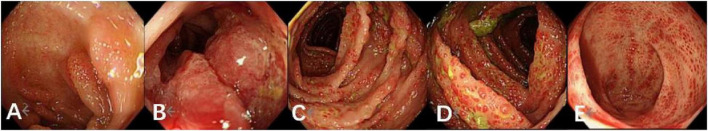
Colonoscopy after admission (2021-01-07). **(A)** Multiple patchy congestion and edema of the mucosa at the terminal part of the stomatch and small intestine. **(B)** A mass of cancerous lesion of the sigmiod colon can be observed 25–30 cm away from the anal verge. **(C–E)** Diffuse patchy congestion of the mucosa from the cecum to the rectum.

**FIGURE 4:**
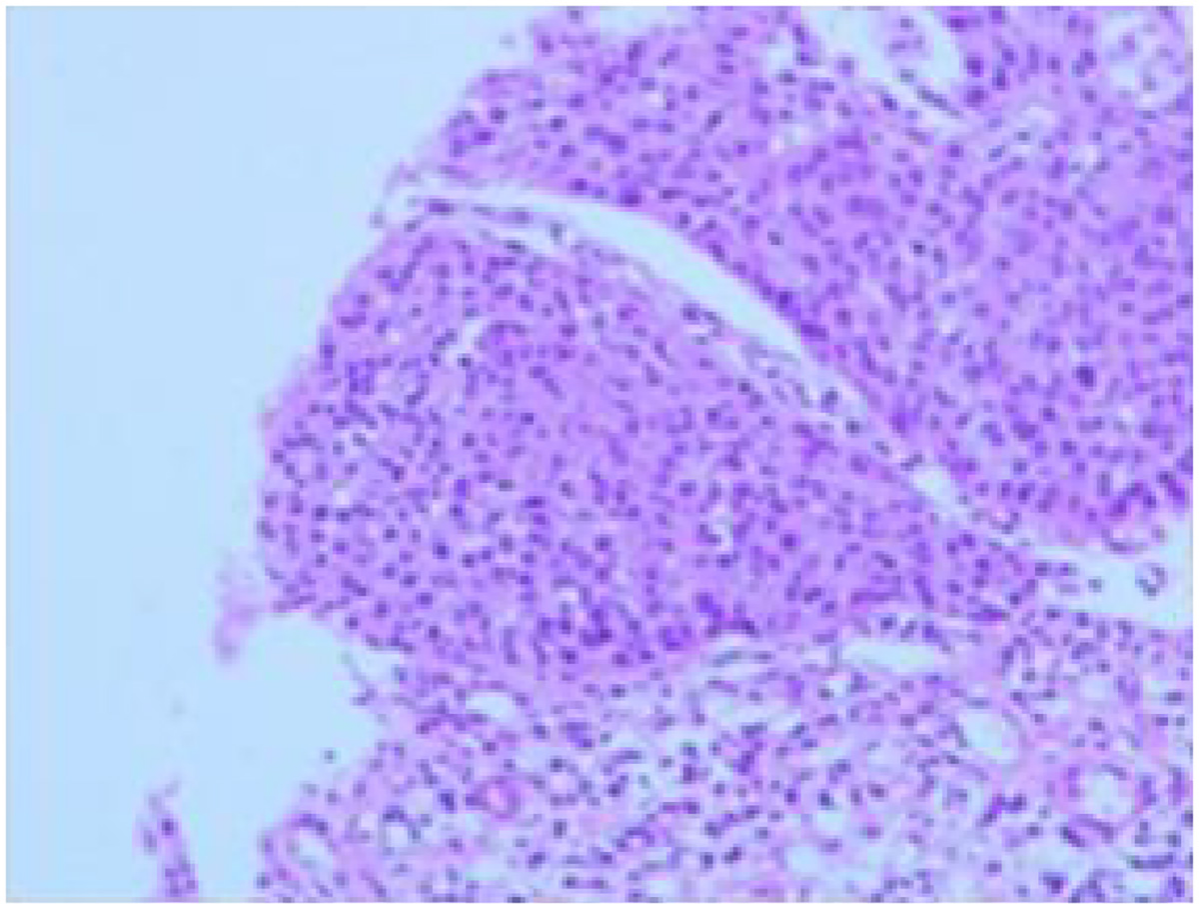
High-grade intraepithelial neoplasia of esophageal squamous epithelium.

**FIGURE 5:**
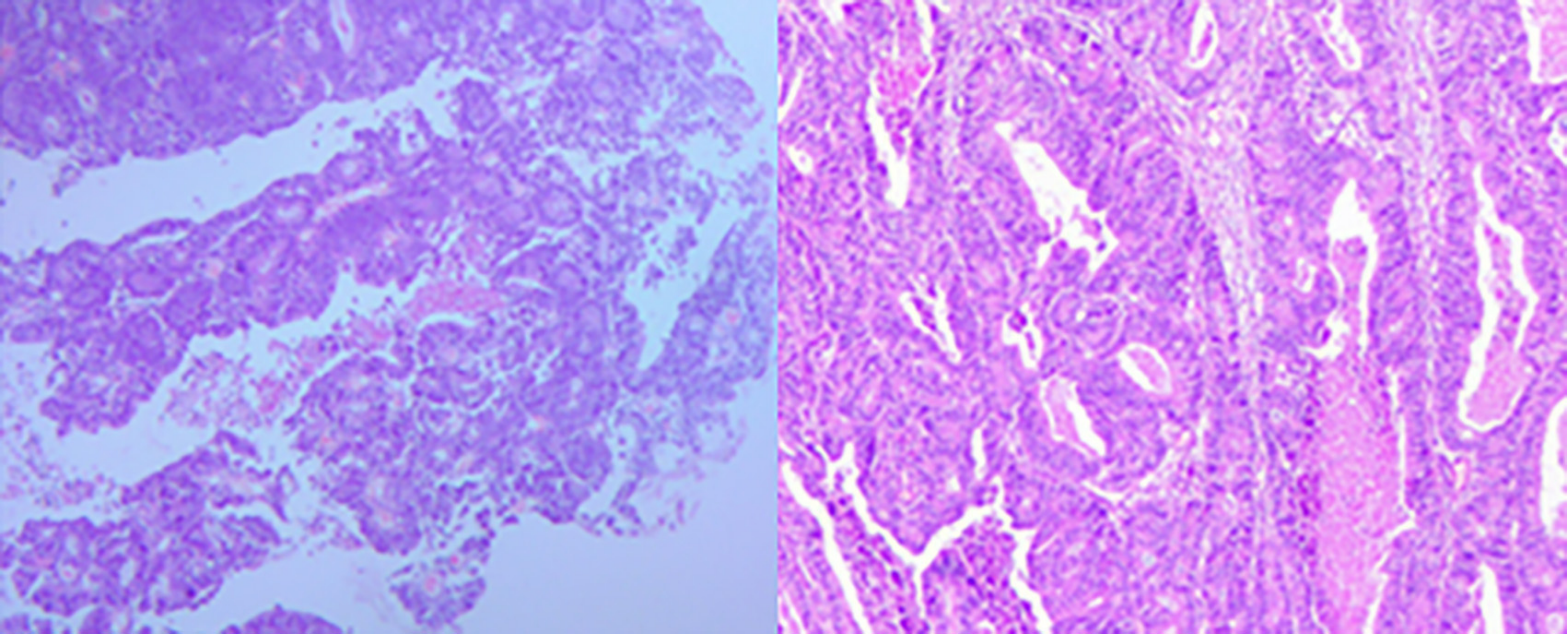
Adenocarcinoma of the sigmoid colon.

After admission, gastroscopy showed (2021-01-07): high-grade intraepithelial neoplasia of the esophageal mucosa, CCS? (Figure 2). Colonoscopy showed (2021-01-07): adenocarcinoma of the sigmoid colon, CCS? (Figure 3). Pathological results: (esophageal) biopsy tissue: moderate chronic inflammation of the mucosa, focal lymphoid tissue hyperplasia in the stroma, partial high-grade intraepithelial neoplasia of squamous epithelium (Figure 4); antral mucosa of the stomach: mild chronic inflammation, interstitial edema with more eosinophilic cell infiltration (> 50 per HPF); (duodenal) biopsy tissue: mild chronic inflammation of the mucosa, interstitial edema with more eosinophilic and neutrophilic cell infiltration (> 50 per HPF); (sigmoid colon) biopsy tissue: adenocarcinoma (Figure 5); (ascending colon and transverse colon) biopsy tissue: moderate chronic inflammation of the mucosa, more eosinophilic and neutrophilic cell infiltration in the stroma, and adenocystic bodies observed. Abdominal and pelvic CT (plain scan and enhanced scan) suggested: significant thickening and enhancement of the sigmoid colon wall, multiple polypoid lesions in the duodenal bulb.

The patient underwent laparoscopic radical resection of sigmoid colon cancer on January 14, 2021. During the operation, the tumor was located approximately 15 cm above the peritoneal reflection, measuring about 4 × 4 × 2 cm, with the capsule infiltrated. The tumor was removed and sent for pathological examination. The postoperative pathology showed: 1. (Sigmoid colon) moderately differentiated adenocarcinoma, invading the muscular layer of the intestinal wall, with suspected nerve infiltration in the lesion, and no clear intravascular cancer emboli. Immunohistochemical staining: CDX-2 (+), MSH2 (+), MSH6 (+), PMS2 (+), P53 (+++), Ki-67 (active area about 70%+), MLH1 (+), CK20 (+), CK7 (−), Villin (+), EGFR (±), E-cadherin (+), S100 (±). 2. No cancer was found at the upper and lower ends. No cancer was found at the mesenteric resection margins. 3. No cancer metastasis was found in regional lymph nodes: 0/17 (beside the intestine), 0/9 (beside the intestine), 0/3 (in the central part of the mesentery), 0/5 (at the root of the mesentery) (Figure 5). Four days after the operation, the patient developed anastomotic fistula and underwent laparoscopic exploration under general anesthesia. During the operation, the intestinal tube in the abdominal cavity was edematous, and the anastomosis was necrotic around the entire circumference. Therefore, laparoscopic sigmoid colostomy was performed, and the patient was discharged on January 26, 2021. On the 5th day after the operation, the patient regularly received “prednisone 50 mg, once a day” treatment, and then the dosage was reduced by 5–10 mg every 1–2 weeks. At the 8th week, the dosage was reduced to 10 mg/day and maintained for 12 months before discontinuation.

**FIGURE 6:**
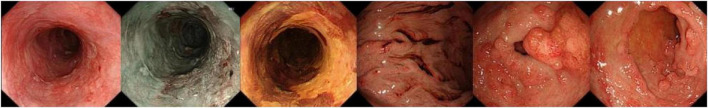
Take prednisone orally for 12 months discontinue for 3 months (2022-04-26)showed: the mucosa on the left and right sides of the esophagus was rough, the NBI showed a tea-brown change, 1.2% Lugol’s iodine staining showed on an unstained area, and the pink sign was positive: the polyps in the gastric body, gastric antrum and duodenum have decreassed compared to before.

**FIGURE 7:**
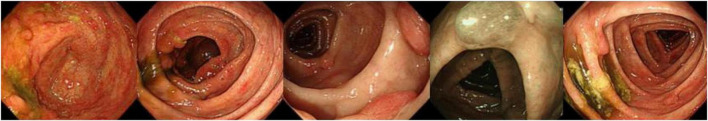
Second colonoscopy re-examination (2022-04-26): Upon inserting the endoscope through the stoma along the lumen, Multiple punctate congestions are observed in the cecal mucosa. Multiple polyps are found from the ascending colon to the stoma.

2022-04-26 Re-examination of gastroscopy: On the left and right walls as well as the posterior wall of the esophagus at a distance of 23–26 cm from the incisors, there was congestion of the mucosa, and the mucosa was rough. Near the focal area, the mucosa showed a tea-brown change and B1-type blood vessels could be seen. 1.2% Lugol’s iodine staining showed a non-stained area and the pink sign was positive. The mucosa of the gastric body, gastric antrum, and duodenum was congested and edematous, and the polyps were less severe than the first time (Figure 6). Gastroscopy diagnosis: Esophageal cancer. Esophageal biopsy pathology: The esophageal mucosa showed severe dysplasia and cancerous changes of squamous epithelium, and no clear infiltration was observed. 2022-04-26 Re-examination of colonoscopy: The scope was inserted 15 cm along the cavity from the anus, and the rectal mucosa was found to be smooth; from the stoma to the cecum, multiple type I and type II polyps with diameters ranging from 0.3 to 0.5 cm of Yamada were observed, with smooth surfaces. Multiple punctate congestion was seen on the cecal mucosa, and multiple type I, II, III and IV polyps with diameters ranging from 0.3 to 1.0 cm were observed from the ascending colon to the stoma, with smooth surfaces (Figure 7). On May 11, 2022, 15 months after the sigmoid colostomy surgery, laparoscopic sigmoid colostomy closure surgery + laparoscopic intestinal adhesion release surgery + colostomy-side hernia repair surgery were performed. After discharge, the patient did not follow the doctor’s advice to treat early esophageal cancer.

**FIGURE 8:**
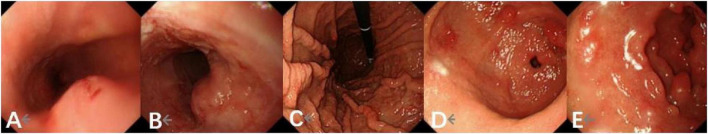
The third review of gastroscopy was performed (2024-08-29). **(A,B)** A neoplastic elevation is observable at a distance of 24 cm from the incisors, infiltrating two-thirds of the esophagus. **(C–E)** The Mucous membrances of the stomach and duodenum are smoother than before, with significantly reduced congestion and edema, and a notable decrease in multiple polyps.

Re-examination of gastroscopy on August 29, 2024: Esophageal cancer. After CCS treatment, compared with the gastroscopy result on January 7, 2021, the mucosa of the stomach and duodenum is smoother, with significant reduction in congestion and edema, and multiple polyps have significantly decreased (Figure 8).

## Discussion

Cronkhite-Canada syndrome is also known as polyp-pigmentation-hair loss-nerve damage syndrome of fingers/toes. It was first discovered by Cronkhite and Canada in 1955 (1). It is a rare non-genetic disease with an incidence rate of approximately one in 1 million (2). Since 1955, only about 500 people worldwide have been diagnosed with CCS (3). The clinical manifestations of CCS mainly include ectodermal changes, digestive tract symptoms, and multiple polyps in the gastrointestinal tract. The ectodermal dysplasia is also known as the triad of skin disorders: (1) hair loss/dehairing occurs in areas such as the scalp, eyebrows, eyelashes, armpits, and limbs; (2) nail malnutrition, which can manifest as atrophy, detachment, and deformity; (3) excessive pigmentation, presenting as brownish patches ranging from light to dark brown distributed on the palms and soles, upper limbs, face, and chest, resembling lentils (4). Gastrointestinal symptoms mainly include chronic diarrhea, loss of appetite, abnormal taste, anemia, abdominal pain, and malnutrition. At present, there is no clear diagnostic standard for CCS. Usually, the following diagnostic factors are used as references: (1) Onset occurs in middle-aged and elderly individuals, without a genetic background, and other hereditary hamartoma polyp disorders have been ruled out; (2) The clinical manifestations often include persistent diarrhea, loss of appetite, and malnutrition; (3) presence of ectodermal changes; (4) endoscopic observation of multiple gastrointestinal polyps; (5) frequent hypoproteinemia. This patient is an elderly male. During the course of the disease, he experienced loss of appetite, diarrhea, vomiting, abnormal taste, and obvious changes in the ectoderm. Gastrointestinal endoscopy reveals typical diffuse polypoid lesions from the stomach, duodenum to the colon. These findings are consistent with the CCS diagnosis.

The cause and mechanism of CCS are currently unclear and may involve multiple factors: Firstly, psychological and physical stress, as well as allergic reactions to substances such as medications or hair dyes, could be the causes of this disease. Reducing exposure to allergens has been found to lower IgE levels and eosinophil infiltration, and to improve clinical symptoms (5). Secondly, patients with CCS often exhibit positive antibodies and high levels of IgG4, suggesting the presence of autoimmune factors (6). The infiltration of IgG4-positive plasma cells into the polyps and the favorable response to immunosuppressive therapy indicate that the autoimmune response is involved in the pathogenesis of CCS (7). CCS is also associated with other autoimmune diseases, such as hypothyroidism, membranoproliferative glomerulonephritis, systemic lupus erythematosus and scleroderma (8). Furthermore, infectious factors also play a role: there is a correlation between CCS and Helicobacter pylori infection, and some patients experienced improvement in symptoms after treatment for Helicobacter pylori (9). Infection with Clostridium difficile (10) and Klebsiella pneumoniae (11) may contribute to the occurrence of CCS. Finally, genetic analysis indicates that mutations in the PRKDC gene may be involved in the pathogenesis of CCS (12), and the inhibition of abnormal expression of the βA gene may also be part of the pathogenesis of CCS (13). In conclusion, it is currently believed that immune abnormalities, bacterial infections, allergic reactions and gene mutations interact with each other to jointly constitute the potential pathogenesis mechanism of this complex disease known as CCS.

A CCS study involving 383 cases in Japan concluded that CCS is a significant risk factor for colon cancer: The prevalence of colon cancer among CCS patients is approximately 10–20%, significantly higher than the prevalence of this type of cancer in the general population (14). The pathological types of CCS polyps include inflammatory polyps, hyperplastic polyps, hamartomatous polyps and adenomatous polyps (15). The pathological manifestation of CCS polyps is characterized by significant infiltration of inflammatory cells such as eosinophils, lymphocytes and neutrophils (16). In addition, abnormal structures of the glands were observed (such as cystic dilation, accompanied by protein fluid or mucus retention in some cases), as well as hyaline membrane edema, as well as edema, congestion and inflammatory glandular hyperplasia of the mucosa between polyps (17). Current research has identified 543 dysregulated genes in CCS colon polyps. These genes are involved in processes such as innate immunity, extracellular matrix disorders, inflammatory cell infiltration, increased angiogenesis, and potential epithelial-to-mesenchymal transition. Further studies on the key overexpressed genes (CXCL1, CXCL3) revealed that chronic inflammation (18) and abnormal proliferation and differentiation of epithelial cells (19) play significant roles in the pathogenesis of CCS digestive tract lesions. The distribution of polyps in patients with CCS is mainly in the stomach and colon (90%). Among them, the antrum of the stomach and the right half of the colon are more common. It may also involve the duodenum and small intestine (80%), the rectum (67%), but is extremely rare in the esophagus (20). There have been reports of cases of CCS combined with esophageal papilloma (21, 22), Esophageal papilloma is mainly formed by abnormal proliferation of esophageal squamous epithelial cells. There has been no previous report of CCS combined with esophageal cancer. In this case, the patient was diagnosed with CCS and at the same time was found to have advanced colon cancer and early-stage esophageal cancer. After 3 years of follow-up, the patient’s early-stage esophageal cancer progressed to advanced esophageal cancer. This patient had no history of alcohol or smoking and no family history of digestive tract cancer. The aforementioned indications of CCS digestive tract lesions are related to chronic inflammation and abnormal proliferation and differentiation of epithelial cells. Therefore, it is worth paying attention to and further exploring whether the coexistence of CCS and early esophageal cancer in this patient is a coincidence or has a common pathogenesis.

If not treated actively, CCS may have a severely poor prognosis due to its complications, with an estimated 5-year mortality rate of up to 55%. With immunosuppressive therapy and nutritional support, the latest reported cases show that the overall 5-year survival rate is 93% (23). After treatment, the clinical symptoms of the patients can be classified into three outcomes: complete clinical remission, partial clinical remission, and no clinical response. Currently, the treatment options for CCS include: glucocorticoids, immunosuppressants, symptomatic and nutritional support, endoscopic treatment, and surgical treatment (24). Among them, corticosteroid therapy, immunosuppressive therapy and nutritional support can improve the natural course of CCS. A foreign study applied an initial prednisone dose of 1 mg/kg/day, gradually reducing the dosage, and usually stopping the medication after 3–6 months. If the hormone dosage was reduced or the condition relapsed after discontinuation, the glucocorticoid dosage could be increased or a new induction remission treatment could be initiated (25). Another study shows that 85% of patients with active CCS who receive hormone therapy at a dose of more than 30 mg per day respond effectively, and usually achieve clinical remission within 12 months (5). Five days after the sigmoid colostomy closure surgery for this patient, after the condition stabilized, prednisone 50 mg was administered once daily for symptomatic and nutritional support treatment. The hormone was used for 12 months, and after discontinuation for 3 months and 2 years and 6 months, the endoscopic reexamination showed that the gastric and duodenal, as well as colonic polypoid lesions had significantly decreased compared to before. The symptoms such as diarrhea, hair loss, and malnutrition were completely relieved clinically. Therefore, immunosuppressive therapy mainly based on glucocorticoids is the most important treatment method for CCS drug therapy. However, there are no unified standards for the initial dose, reduction plan, and duration of maintenance medication. Moreover, at present, there is still some uncertainty regarding the long-term prognosis (5-year survival rate, recurrence pattern, and quality of life) of patients with double cancer resection who have undergone CCS. In the future, clinicians need to conduct comprehensive assessments based on the patient’s condition, provide regular and long-term follow-up, and offer health education. They should encourage patients to actively undergo endoscopic monitoring. Moreover, multi-center and prospective cohort studies can be conducted for follow-up to further clarify the optimal treatment sequence, auxiliary treatment plans, and prognostic influencing factors, thereby optimizing clinical decisions.

In conclusion, this CCS case was accompanied by sigmoid colon cancer and early esophageal cancer. After surgical treatment combined with hormone therapy, the colon cancer and CCS achieved clinical remission. Unfortunately, due to the presence of advanced colon cancer and the patient’s insufficient attention to early esophageal cancer, the clinical treatment focus shifted primarily to radical surgery and adjuvant therapy for colon cancer, and the endoscopic treatment for early esophageal cancer was refused. As a result, the early esophageal cancer progressed. This article summarizes and reports the diagnosis and treatment process of this case, with the aim of enhancing clinicians’ understanding of CCS. At the same time, the mechanism and clinical management of CCS combined with colon cancer and other digestive tract tumors also warrant in-depth research and discussion.

## Data Availability

The original contributions presented in the study are included in the article/Supplementary material, further inquiries can be directed to the corresponding author.
